# Nutritional anthropometric indicators predict achievable pocket depth in totally implantable venous access port implantation: a prospective study with 3D-printed measurement device

**DOI:** 10.3389/fonc.2026.1898363

**Published:** 2026-10-22

**Authors:** Min Hu, Xiangrui Chen, Xiaomei He, Chengluo Hao, Xiaobo Tong, Yunwei Han

**Affiliations:** 1 Department of Dermatology, Zigong Third People’s Hospital, Zigong, Sichuan, China; 2 Department of Oncology, Zigong Third People’s Hospital, Zigong, Sichuan, China; 3 Department of Oncology, Affiliated Traditional Chinese Medicine Hospital of Southwest Medical University, Luzhou, China

**Keywords:** 3D printing, abdominal fat, nutritional status, prospective study, totally implantable venous access port

## Abstract

**Background:**

Pocket depth is a critical anatomical factor affecting complications after totally implantable venous access port (TIVAP) implantation. However, intraoperative depth assessment relies on empirical estimation without objective quantification. The relationship between preoperative nutritional status and achievable pocket depth remains unclear.

**Methods:**

This prospective single-arm clinical trial enrolled 37 patients undergoing TIVAP implantation assisted by a novel 3D-printed pocket depth measurement device between September 2025 and February 2026. The primary endpoint was device-related adverse events (DRAEs) at 30-day follow-up. Secondary endpoints included the correlation between nutritional anthropometric indicators (max abdominal fat thickness by CT, waist circumference, BMI) and pocket depth.

**Results:**

No device-related adverse events occurred (0%, 95% CI 0%-8.1%). Total complication rate was 2.7% (1/37). Max abdominal fat thickness demonstrated the strongest correlation with pocket depth (*r* = 0.803, 95% CI 0.64-0.89, *p* < 0.001), explaining 64.4% of variance (R^2^= 0.644). The prediction equation was: Pocket Depth (mm) = 2.50 + 0.103 × Max Abdominal Fat Thickness (mm). ROC analysis, following 1,000-iteration bootstrap internal validation, showed excellent predictive accuracy for deep pocket (≥5mm) with an optimism-corrected AUC of 0.942 (95% CI: 0.860-1.000) and an optimal cut-off of 14.5 mm (sensitivity 91.3%, specificity 85.7%). Notably, 21.6% of patients (8/37) had abdominal fat <10mm and achievable depth ≤3mm despite surgical goal of ≥5mm, indicating anatomical limitation imposed by subcutaneous tissue availability.

**Conclusion:**

In the context of an early feasibility study, this research demonstrated the preliminary safety and feasibility of a 3D-printed pocket depth measurement device for TIVAP implantation. It pioneers the quantification of a strong correlation between nutritional indicators and pocket depth, enabling the proposal of exploratory risk stratification thresholds to identify patients with anatomical limitations theoretically associated with shallow pocket depth, and offering a novel nutritional perspective for subcutaneous device implantation.

## 1 Introduction

Totally implantable venous access port (TIVAP) has become the standard vascular access device for long-term chemotherapy, nutritional support, and blood transfusion therapy in patients with malignant tumors (1, 2). Compared with peripheral venous catheters, TIVAP offers advantages including lower infection risk, longer maintenance intervals, and improved quality of life (3). However, postoperative complications following TIVAP implantation remain a major clinical challenge. Recent systematic reviews have shown that the incidence of TIVAP-related complications ranges from 1.8% to 14.4%, with port extrusion, poor wound healing, and catheter-related infection being the most common late complications (4). Existing evidence indicates that pocket depth is a critical anatomical factor affecting complication occurrence: excessively shallow depth may lead to port compression against the skin causing ischemic necrosis and extrusion, while excessive depth increases procedural difficulty and hematoma risk. Although clinical consensus recommends a pocket depth of ≥5 mm to ensure adequate soft tissue coverage, in actual surgical practice, operators often rely on empirical visual estimation of depth without objective quantification standards, resulting in significant individual variability.

The nutritional status of cancer patients exhibits high heterogeneity, ranging from cachexia to obesity, which directly affects subcutaneous tissue thickness and surgical anatomical conditions. The European Society for Clinical Nutrition and Metabolism (ESPEN) guidelines emphasize that accurate nutritional assessment is crucial for treatment decision-making in cancer patients (5). Traditional nutritional assessment indicators such as body mass index (BMI) cannot distinguish between muscle and fat distribution, whereas computed tomography (CT)-based body composition analysis has been confirmed to more accurately reflect patients’ fat reserves and nutritional risk (6). However, no study has yet established a quantitative association between preoperative nutritional anthropometric indicators (such as abdominal wall fat thickness and waist circumference) and the anatomical limitations of TIVAP pocket depth. Clinically, patients with poor nutritional status are often encountered, and even with optimal surgical techniques, the achievable pocket depth may be limited to 2–3 mm. Whether such patients belong to the high-risk population for port extrusion still lacks evidence-based medical support.

With the development of medical additive manufacturing technology, 3D printing technology has provided new approaches for customized surgical assistive tools (7, 8). Previous studies have confirmed the value of 3D-printed guides in improving surgical precision, but their application in the field of venous access remains blank. Based on this, our research team independently developed a medical-grade 3D-printed pocket depth measurement device aimed at achieving objective quantification of intraoperative depth. This study intends to conduct a prospective single-arm clinical trial to first evaluate the safety and feasibility of the measurement device; secondly, to focus on exploring the correlation between preoperative nutritional anthropometric indicators (particularly CT-measured maximum abdominal fat thickness) and achievable pocket depth, and to establish a nutrition-based depth prediction model. This study not only provides a measurement tool for TIVAP implantation but also reveals the association of subcutaneous tissue reserves with surgical anatomical limitations from the perspective of nutritional anthropometry, providing a basis for preoperative planning.

## 2 Methods

### 2.1 Study design

This prospective, single-arm clinical study was conducted at the Department of Oncology, Zigong Third People’s Hospital, Sichuan Province, China, from September 2025 to February 2026. The research protocol was rigorously reviewed and approved by the Institutional Medical Ethics Committee (Approval No. IEC-AF/SS[Research]-03-2.0). This study was funded by the “Key Science and Technology Program of Zigong - Zigong Medical Big Data and Artificial Intelligence Research Institute Collaborative Innovation Category Project” (Grant No. 2025YGY0321). All procedures adhered to the principles of the Declaration of Helsinki and China’s “Good Clinical Practice Guidelines for Drugs” (GCP).

This study aimed to evaluate the safety and clinical practicality of applying a 3D-printed pocket depth measurement device during TIVAP implantation. As a first-in-human exploratory trial, the primary objective was to assess the device-related safety of the measurement device. The secondary objective was to explore the correlation between pocket depth and nutritional anthropometric indicators (such as maximum abdominal fat thickness, waist circumference, and BMI). The sample size was determined according to the FDA Early Feasibility Medical Device Clinical Studies guidance, which recommends enrolling 20–40 patients to identify device-related adverse events with an incidence rate ≥10%. This study ultimately enrolled 37 patients, meeting the ethical requirements and statistical power for first-in-human research. The calculation was based on the historical incidence of complications (1.8%-14.4%) in previous TIVAP-related studies, using a one-sided test to ensure identification of clinically significant safety signals. An independent Data Safety Monitoring Board was established during the study to periodically review safety data and had the authority to recommend early study termination based on evaluation results. All patients signed written informed consent before enrollment, confirming their understanding of the treatment content, potential risks, alternative options, and the right to withdraw at any time without affecting subsequent treatment. The study flowchart is shown in **Figure 1**.

**Figure 1:**
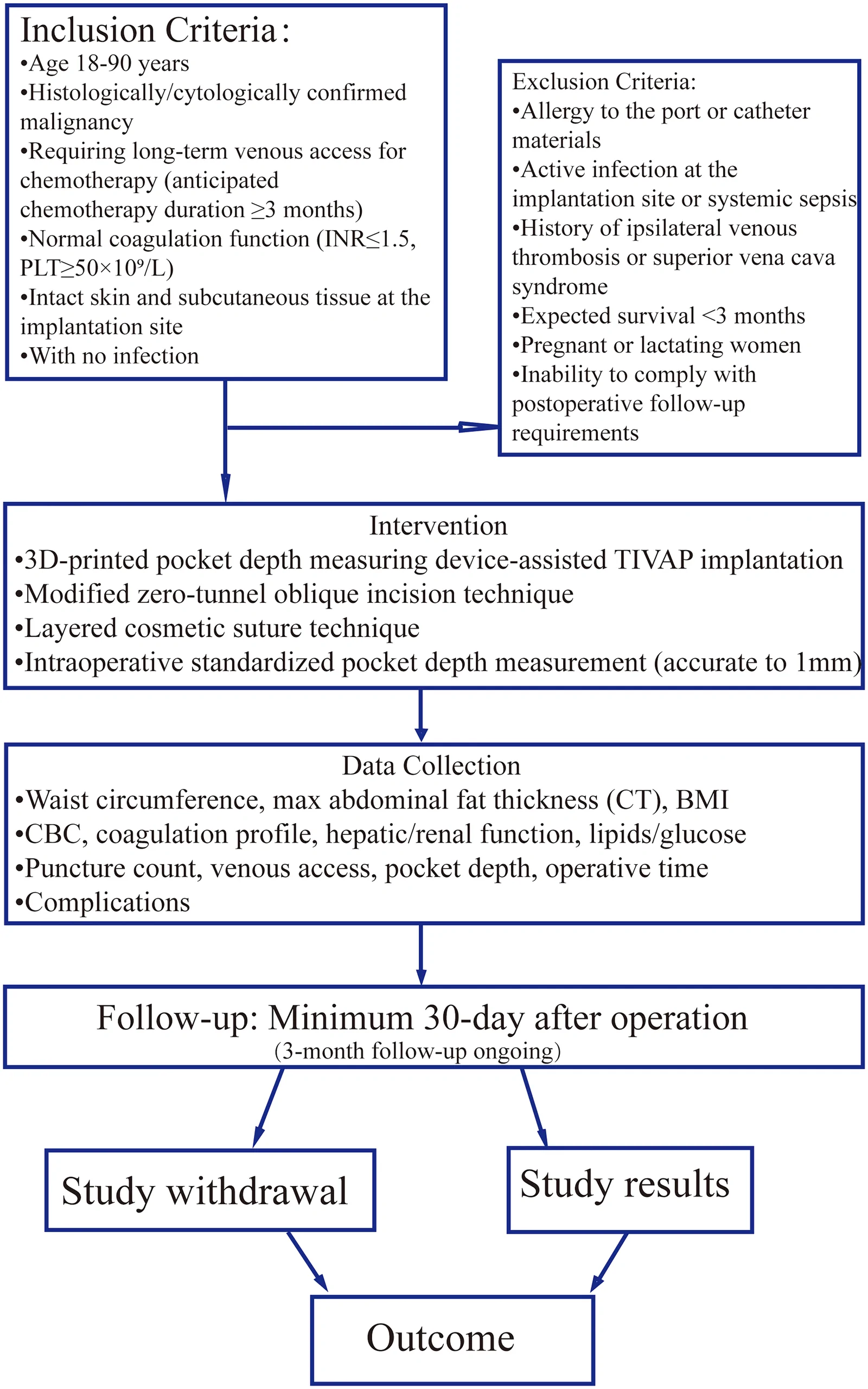
Study flowchart of the prospective single-arm trial. Thirty-seven patients undergoing totally implantable venous access port (TIVAP) implantation assisted by a 3D-printed pocket depth measurement device were enrolled between September 2025 and February 2026 at Zigong Third People’s Hospital. All patients completed a minimum 30-day follow-up with no loss to follow-up. The primary safety endpoint was assessed at 30 days, while 3-month follow-up data are ongoing. Patients were analyzed for device safety, procedural feasibility, and the correlation between nutritional anthropometric indicators and pocket depth.

### 2.2 Patient selection

Inclusion criteria included: (1) age 18–90 years; (2) histologically or cytologically confirmed malignant tumor; (3) clinical assessment requiring long-term venous chemotherapy access (expected chemotherapy duration ≥3 months); (4) normal coagulation function (INR ≤1.5, platelet count ≥50×10^9^/L; (5) intact skin and subcutaneous tissue at the implantation site without infection. Exclusion criteria included: (1) allergy to port or catheter materials; (2) active infection at the implantation site or systemic sepsis; (3) history of ipsilateral venous thrombosis or superior vena cava syndrome; (4) expected survival <3 months; (5) pregnant or lactating women; (6) inability to comply with postoperative follow-up requirements.

Before the study began, GCP-trained researchers explained the study purpose, procedures, potential risks and benefits, alternative treatment options, and subject rights to each potential participant in detail. All patients signed written informed consent after full understanding and had the right to withdraw from the study unconditionally at any stage without affecting subsequent routine medical care. All collected data were anonymized and used only for this study analysis.

### 2.3 The 3D-printed depth measurement device

The pocket depth measurement device independently developed by our research team used computer-aided design (CAD) software for three-dimensional modeling and structural optimization (**Figure 2A**). The main body of the device was manufactured using stereolithography (SLA) 3D printing technology, selecting medical-grade biocompatible photosensitive resin material that has passed biocompatibility certification to ensure safety for contact with human subcutaneous tissue. The overall design adopted a compact handheld structure, facilitating single-hand operation by the surgeon within the sterile field while avoiding interference with the surgical field (**Figure 2B**). The device surface underwent post-curing and polishing treatment to reduce surface roughness, prevent bacterial hiding, and minimize mechanical friction damage to subcutaneous tissue.

**Figure 2:**
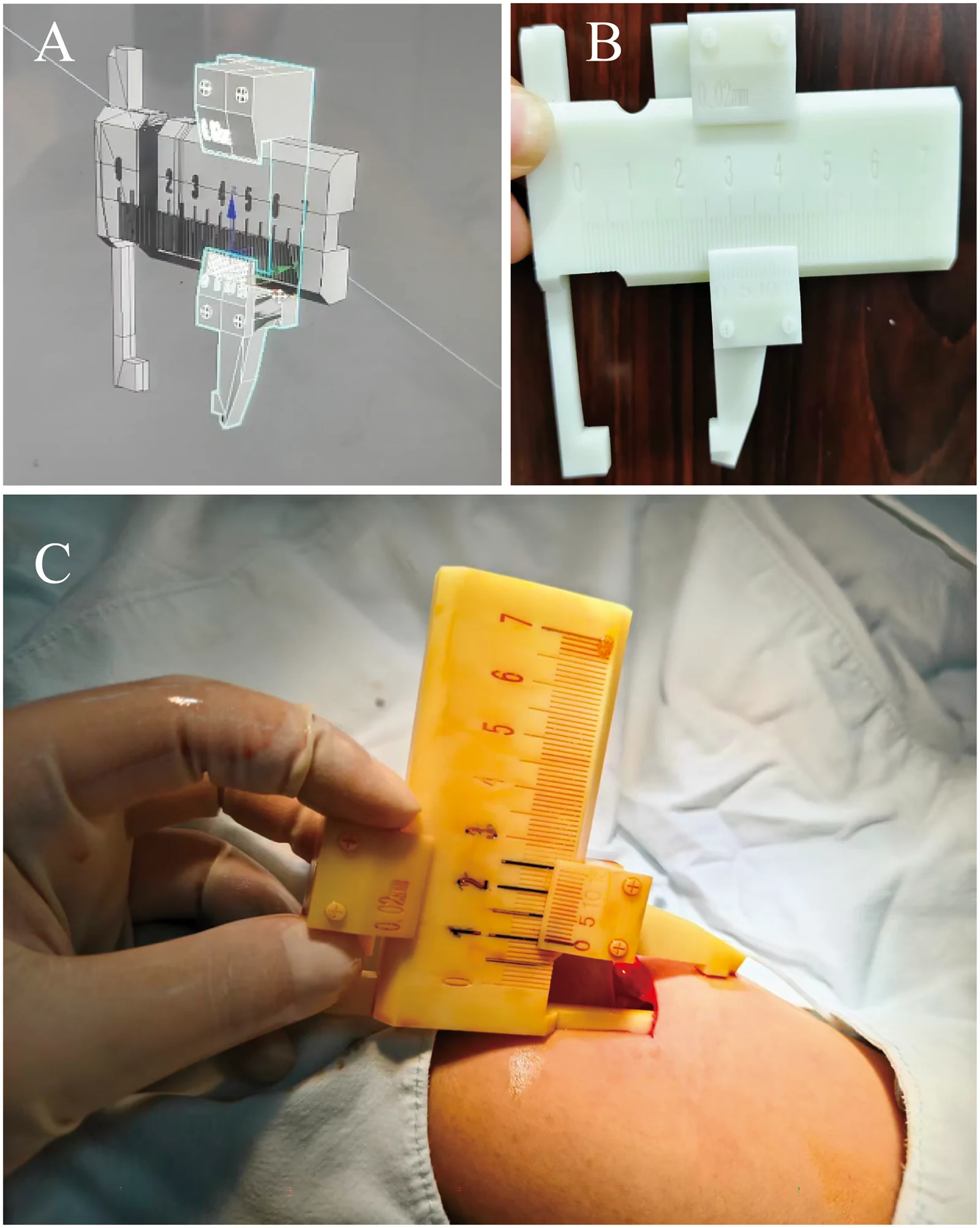
Design, fabrication, and intraoperative application of the novel 3D-printed pocket depth measurement device. **(A)** shows the computer-aided design (CAD) three-dimensional model with caliper-like structure. **(B)** displays the fabricated device produced via stereolithography (SLA) 3D printing using medical-grade biocompatible photopolymer resin (USP Class VI certified), with measurement precision of 1mm and verified accuracy within ±0.5mm. **(C)** demonstrates intraoperative standardized pocket depth measurement after pocket creation and before port implantation, with the probe inserted vertically to read the distance from skin surface to pocket base. The device undergoes high-level disinfection with 10% povidone-iodine immersion for 30 minutes before each use, and the average measurement time is approximately 20–30 seconds.

The measurement device was designed based on the vernier caliper principle, consisting of two parts: a main scale and a sliding probe, with a scale precision of 1 mm. Before putting into use, the research team used standard precision vernier calipers to verify the accuracy of the 3D-printed device. By measuring standard blocks of different thicknesses (1–10 mm), it was confirmed that the measurement error was controlled within ± 0.5 mm, meeting the requirements for clinical precise measurement. To address interobserver reliability, all intraoperative measurements in this study were strictly performed by a single senior surgeon following a standardized protocol to minimize variability. However, we acknowledge that this approach does not establish formal interobserver reliability, which represents a methodological limitation. Compared with traditional intraoperative visual estimation, the device can provide objective and quantitative depth data, effectively reducing pocket depth variability caused by operator experience differences.

Given that the device was designed as a reusable instrument, this study formulated a strict high-level disinfection protocol. However, SLA medical photosensitive resin materials have thermal sensitivity with a low glass transition temperature and cannot withstand conventional high-temperature and high-pressure sterilization (such as autoclave steam sterilization or dry heat sterilization); otherwise, it would lead to material softening, structural deformation, and loss of measurement accuracy. Therefore, this study selected chemical disinfection, specifically using 10% povidone-iodine solution immersion disinfection. This disinfectant has broad-spectrum antibacterial activity and has been verified to have good chemical compatibility with medical resin materials, without corroding the scale or changing the mechanical properties of the material. Before each surgery, the device was completely immersed in the disinfectant solution for 30 minutes, followed by thorough rinsing with sterile saline 3 times to remove residual iodine. To ensure disinfection effectiveness and material stability and to rigorously demonstrate the adequacy and safety of this chemical disinfection for a reusable device, validation experiments were conducted in the early stage of the study: printed samples were cultured for bacteria after disinfection following the above protocol 3 times, and all results were negative; meanwhile, after the samples underwent 20 repeated disinfection cycles, precision testing was performed again using standard blocks, and no significant deformation or increased scale error was found (error controlled within ±0.05 mm), proving that this protocol achieves adequate high-level disinfection and microbiological safety while maintaining good material tolerance and structural stability. Although chemical disinfection cannot achieve absolute sterility (as defined by autoclaving), it is the only viable option for this heat-sensitive resin. Furthermore, this measurement device is not implanted; it is used briefly (20–30 seconds) within the subcutaneous pocket, and the pocket is subsequently irrigated with 10–20 ml of 10% povidone-iodine solution before port insertion, providing a dual barrier against infection. Despite these validation results, we acknowledge several limitations of this chemical disinfection protocol (1): it relies on proper implementation and adherence to the 30-minute immersion time (2); long-term material degradation beyond 20 cycles was not evaluated (3); the protocol has not been validated against microbiological endpoints in clinical use. Future studies should evaluate infection rates associated with device reuse.

The intraoperative measurement timing was standardized as after pocket creation and before port implantation. The operator inserted the measurement device probe vertically into the bottom of the pocket and read the vertical distance from the skin surface to the bottom of the pocket, with values accurately recorded to 1 mm (**Figure 2C**). If the measured depth deviated from the preset target value, the operator could immediately adjust the pocket dissection layer until the ideal depth was achieved. All measurement data were recorded in real-time into the electronic case report form (eCRF) as core variables for postoperative analysis of the association between pocket depth and complications. The entire measurement process took an average of approximately 20–30 seconds and did not additionally increase the total surgical time.

### 2.4 Surgical procedure and depth measurement

All surgeries were completed by a fixed tumor interventional team (including 1 tumor interventional physician with more than 5 years of interventional surgery experience, 1 dermatological cosmetic physician, 2 tumor interventional nurses, and 1 radiological interventional technician) under standard sterile operation. Based on safety considerations that chest wall ports may cause pneumothorax, the surgical site prioritized arm port selection; chest wall port was performed only if the arm venous vessels were too thin or arm venous puncture failed. Venous access establishment selected different guidance methods according to port type: arm ports used ultrasound-guided puncture, preferentially selecting the basilic vein; if basilic vein puncture failed or vasospasm occurred, the cephalic vein or ipsilateral subclavian vein was attempted sequentially; chest wall ports used DSA-guided puncture, preferentially selecting the subclavian vein, and if necessary, changing to ultrasound-guided internal jugular vein puncture to ensure successful access establishment. Puncture attempts and venous access establishment conditions were recorded intraoperatively.

Specific surgical procedure: The patient was placed in the supine position, the surgical area was routinely disinfected and draped, and 2 ml of 1% lidocaine was used for local infiltration anesthesia at the puncture site. Then ultrasound-guided venous puncture was performed; if puncture was unsuccessful after 2 attempts, the puncture site was changed. After successful puncture, a guide wire was inserted through the puncture needle, then a catheter introducer sheath was placed over the guide wire (**Figure 3A**), and under DSA guidance, the TIVAP catheter was advanced through the introducer sheath into the vein until its tip reached the right atrium. Then 15 ml of 1% lidocaine was used for local anesthesia in the cutting area and pocket area (**Figure 3B**). Starting from the puncture point, an oblique incision of approximately 45° with a length of about 2.5 cm was made, and tissue was cut layer by layer using electrocautery (**Figure 3C**), with a cutting depth of approximately 6 mm. In the opposite direction of catheter insertion, subcutaneous tissue was carefully dissected using a combination of blunt and sharp methods to create a subcutaneous pocket of appropriate size, trying to ensure that the pocket depth was not less than 4 mm; for emaciated patients with less subcutaneous fat, the bottom of the pocket should adhere to the fascia on the muscle surface. Different from the traditional double-incision technique, this protocol does not require additional subcutaneous tunnel creation; the catheter is directly introduced into the pocket through the incision, reducing subcutaneous tissue injury and tunnel-related complication risks. After pocket creation was completed, the operator confirmed again that there was no active bleeding in the pocket, then used the standard disinfected pocket depth measurement device for depth measurement and recording (**Figure 3D**); the entire measurement process took an average of approximately 20–30 seconds. After measurement, 10–20 ml of 10% povidone-iodine solution was used to rinse the pocket. The excess part of the catheter end was cut off, then connected to the port, the safety valve was locked, and after confirming smooth blood withdrawal, the pipeline was flushed with 100 U/ml heparin saline and positive pressure sealing was performed, then the port was pushed into the pocket. After DSA or ultrasound confirmed again that the TIVAP and catheter positions were correct, a non-injury needle was inserted at the TIVAP site, and 4–0 absorbable suture was used for careful subcutaneous tissue tension-reduction suturing to ensure moderate tissue coverage thickness and uniform tension on the port surface; if necessary, intradermal suturing could also be adopted (**Figure 3E**) to ensure that surgical incision tension was minimized as much as possible; subsequently, 6–0 absorbable suture was used for interrupted intradermal suturing to align the skin edges (**Figure 3F**). The incision was covered with sterile dressing, tension-reduction tape was used as appropriate, and appropriate pressure bandaging was applied.

**Figure 3:**
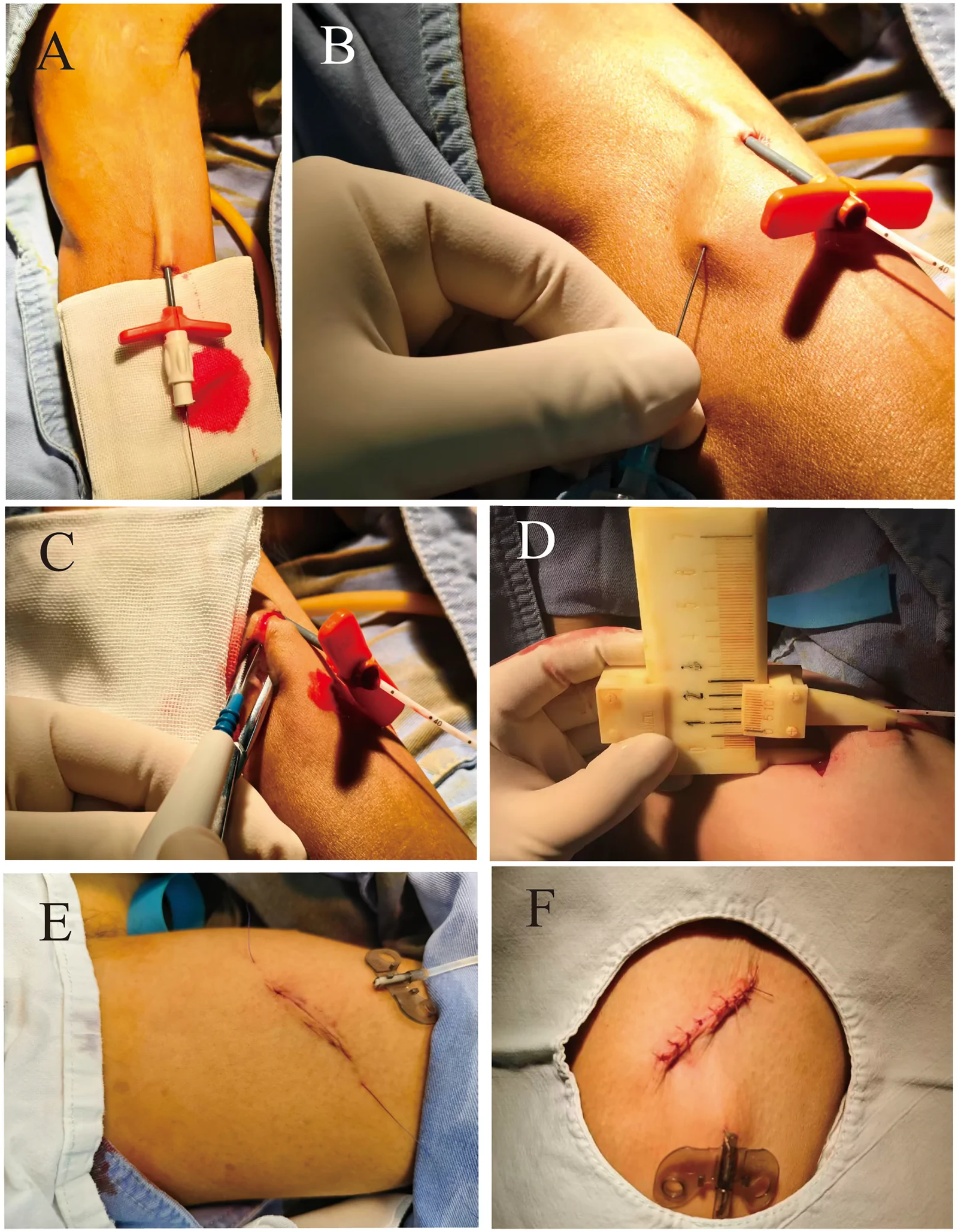
Intraoperative photographs illustrating the key steps of 3D-printed device-assisted TIVAP implantation with modified zero-tunnel oblique incision technique. **(A)** Insertion of the catheter introducer sheath over the guidewire under ultrasound guidance. **(B)** Local infiltration anesthesia with 1% lidocaine (15ml) at the pocket creation site. **(C)** Oblique incision (approximately 2.5cm in length, 45° angle) made from the puncture site using electrocautery. **(D)** Standardized pocket depth measurement using the 3D-printed device after pocket creation and before port implantation. **(E)** Subcutaneous tension-reduction sutures with 4–0 absorbable suture for arm port. **(F)** Intradermal interrupted sutures with 6–0 absorbable suture for arm port. The modified zero-tunnel technique eliminates the need for a separate tunnel incision, reducing subcutaneous tissue trauma. Written informed consent was obtained from all patients for publication of these intraoperative photographs.

After surgery, the operating physician recorded puncture and incision-related conditions, including puncture attempts, pocket depth, and perioperative complications.

### 2.5 Follow-up plan and evaluation metrics

This study formulated a standardized postoperative follow-up plan, with the primary safety endpoint being 30 days postoperatively; 3-month follow-up is still ongoing. Follow-up methods included a combination of outpatient review and telephone follow-up, executed by uniformly trained research assistants. Follow-up content mainly focused on TIVAP functional status, incision healing conditions, and complication monitoring. If patients experienced any suspected catheter-related adverse events during the follow-up interval, unscheduled follow-up was initiated and recorded in time. All follow-up data were recorded in real-time into the eCRF to ensure data integrity and traceability. The final follow-up rate of this study was 100%, with no loss to follow-up cases, providing a reliable basis for safety assessment.

Preoperative anthropometric indicators and laboratory indicators, as the core exposure variables of this study, were collected within 48 hours before surgery. Anthropometric indicators included: (1) waist circumference: patients stood upright, and a non-elastic tape measure was used to measure the circumference at the level between the lower rib margin and the iliac crest midpoint, accurate to 0.1 cm; (2) maximum abdominal fat thickness: abdominal CT scanning was performed to identify the thickest abdominal wall fat layer, and the fat thickness at that location was accurately measured, accurate to 0.1 mm; (3) height and weight: standardized height and weight instruments were used for measurement, and body mass index (BMI, kg/m^2^) was calculated. Laboratory indicators included blood routine (WBC, HB, PLT), coagulation function (PT, INR), liver function (ALT, AST, TBil, ALB), and blood lipids and glucose, etc.; all data were extracted from the hospital electronic medical record system (EMR).

Safety assessment was the primary endpoint of this study, and complication monitoring covered two core time windows: perioperative and 30 days postoperatively. Perioperative complications included puncture failure, puncture site bleeding (>5 ml), pocket bleeding (>5 ml), pneumothorax, air embolism, arrhythmia, etc., recorded in real-time by the operator intraoperatively. Postoperative early complications were defined according to international TIVAP consensus standards, with monitoring ending at 30 days postoperatively, including catheter-related infection, venous thrombosis formation, hematoma, port extrusion, poor wound healing, catheter fracture or detachment, port rotation, and dysfunction, etc. All suspected complications were recorded as binary variables (occurred/not occurred). If complications occurred, an independent endpoint adjudication committee performed causal relationship determination to distinguish whether they were device-related adverse events (DRAEs). Given that this study was a medical device early feasibility trial, the focus was on identifying safety signals; therefore, complication incidence rates were mainly reported. Postoperative long-term follow-up (3 months and above) is still ongoing, aiming to evaluate late complications; relevant data will be reported separately in subsequent studies. This study mainly reported safety data within 30 days postoperatively to ensure that all enrolled patients reached the minimum follow-up time limit, guaranteeing data integrity and authenticity.

### 2.6 Statistical analysis

All statistical analyses were completed using SPSS 26.0 software. After normality assessment of continuous variables using the Shapiro-Wilk test, data conforming to normal distribution were expressed as mean ± standard deviation ( $\bar{x}$ ± s), and between-group comparisons used independent sample t-test; non-normally distributed data were expressed as median (interquartile range) [M(IQR)], and between-group comparisons used Mann-Whitney U test. Categorical variables were expressed as frequency (percentage), and between-group comparisons used χ^2^ test or Fisher’s exact test (when theoretical frequency <5). The core analysis of this study was the correlation assessment between pocket depth and nutritional anthropometric indicators; Pearson correlation coefficient (r) was used to quantify the linear correlation intensity between pocket depth and abdominal fat thickness, waist circumference, and BMI, and 95% confidence intervals (95% CI) were calculated. To establish a depth prediction model, simple linear regression analysis was used, with pocket depth as the dependent variable and nutritional indicators as independent variables, reporting regression coefficient (β), standard error (SE), and coefficient of determination (R^2^). Receiver operating characteristic (ROC) curve analysis was used to evaluate the discriminative ability of nutritional indicators for predicting deep pocket (≥5 mm), and the Youden index was used to determine the optimal diagnostic cut-off value, reporting AUC, sensitivity, and specificity. To address potential overfitting concerns related to the small sample size, internal validation was performed using 1,000 iterations of bootstrap resampling. For each iteration, the dataset was resampled with replacement to create a bootstrap sample of the same size. The apparent AUC was calculated on this bootstrap sample, and the test AUC was calculated on the out-of-bag (OOB) observations. Optimism was defined as the difference between the mean apparent AUC and the mean OOB test AUC across all iterations. The optimism-corrected AUC was then calculated by subtracting this estimated optimism from the original apparent AUC of the full dataset, along with its 95% confidence interval.

Safety analysis used descriptive statistical methods; the incidence rates of DRAEs and TIVAP-related complications were expressed as percentages, and the 95% confidence interval for zero event incidence rate was calculated using the “Rule of Three” (i.e., 95% CI upper limit ≈ 3/n, which equals 8.1% for n=37). Subgroup analyses were conducted by gender (male/female) and implantation site (arm port/chest wall port) to evaluate the safety consistency of the measurement device in different populations. Missing data used complete case analysis, including only 37 patients with all key variables complete. All statistical tests were two-sided, and P <0.05 was considered statistically significant; this study did not perform multiple comparison corrections because the main analysis was a pre-set univariate correlation test.

## 3 Results

### 3.1 Baseline characteristics of participants

As shown in **Table 1**, a total of 37 patients undergoing TIVAP implantation using a novel 3D-printed depth measurement device were enrolled in this study between September 2025 and February 2026. The cohort was predominantly male (67.6%), with a mean age of 63.9 ± 12.8 years. Most patients received arm ports (81.1%), while 18.9% of patients underwent chest wall port implantation. Although patients had underlying malignancies requiring chemotherapy, most patients had stable vital signs (systolic blood pressure 117.8 ± 24.4 mmHg, diastolic blood pressure 73.8 ± 9.3 mmHg) and sufficient baseline physical status suitable for surgical intervention.

**Table 1:** Baseline characteristics of patients undergoing totally implantable venous access port placement with 3D-printed depth measurement device.

Variable	Value [mean ± SD or n (%)]
Gender	
Male	25 (67.6%)
Female	12 (32.4%)
Age (years)	63.9 ± 12.8
Systolic blood pressure (mmHg)	117.8 ± 24.4
Diastolic blood pressure (mmHg)	73.8 ± 9.3
White blood cell count (×10^9^/L)	6.6 ± 2.1
Hemoglobin (g/L)	122.2 ± 19.0
Platelet count (×10^9^/L)	203.2 ± 103.4
Prothrombin time (s)	11.3 ± 1.0
INR	1.0 ± 0.1
D-dimer (mg/L)	1.1 ± 1.4
Alanine aminotransferase (U/L)	24.7 ± 19.0
Aspartate aminotransferase (U/L)	24.4 ± 10.3
Total bilirubin (μmol/L)	10.7 ± 3.5
Albumin (g/L)	38.1 ± 10.5
Blood urea nitrogen (mmol/L)	5.6 ± 1.8
Creatinine (μmol/L)	65.6 ± 21.2
Glucose (mmol/L)	6.4 ± 1.8
Triglyceride (mmol/L)	1.5 ± 0.8
Total cholesterol (mmol/L)	4.8 ± 1.0
Height (cm)	162.1 ± 8.3
Weight (kg)	56.4 ± 8.0
Body Mass Index (kg/m^2^)	21.5 ± 3.3
Waist circumference (cm)	84.4 ± 9.2
Max abdominal fat thickness (mm)	19.2 ± 11.1
Port type	
Arm port	30 (81.1%)
Chest wall port	7 (18.9%)

Baseline demographic, clinical, laboratory, and anthropometric characteristics of 37 patients undergoing totally implantable venous access port (TIVAP) placement with a novel 3D-printed pocket depth measurement device. Data are presented as mean ± standard deviation or number (percentage). Max abdominal fat thickness was measured using CT scanning of the abdomen. The thickest layer of abdominal wall fat was identified, and the fat thickness at this location was precisely measured to the nearest 0.1 mm. INR, international normalized ratio.

The baseline organ function of the cohort patients was generally well maintained. Hematological parameters showed mild anemia in some cases (hemoglobin 122.2 ± 19.0 g/L), but platelet count (203.2 ± 103.4 × 10^9^/L) and white blood cell count (6.6 ± 2.1 × 10^9^/L) remained within normal ranges. Coagulation function indicators were at normal levels (prothrombin time 11.3 ± 1.0 s, INR 1.0 ± 0.1), ensuring procedural safety. Liver and kidney function tests showed sufficient reserve: alanine aminotransferase was 24.7 ± 19.0 U/L, albumin was 38.1 ± 10.5 g/L, and creatinine was 65.6 ± 21.2 μmol/L. Metabolic parameters including glucose (6.4 ± 1.8 mmol/L) and blood lipids (triglyceride 1.5 ± 0.8 mmol/L, total cholesterol 4.8 ± 1.0 mmol/L) also remained stable.

To evaluate the correlation with pocket depth, we meticulously recorded anthropometric data. The mean body mass index was 21.5 ± 3.3 kg/m^2^, and the mean waist circumference was 84.4 ± 9.2 cm. Notably, the maximum abdominal fat thickness measured by CT scanning averaged 19.2 ± 11.1 mm, showing considerable variability among different patients. This cohort represents a group of strictly screened patients who required long-term venous access due to malignant tumors but retained liver and coagulation function. These baseline characteristics reflect real-world clinical practice, namely selecting patients with sufficient physiological reserves for TIVAP implantation to facilitate smooth implementation of subsequent chemotherapy regimens.

### 3.2 Safety and complication outcomes

Safety outcomes are summarized in **Table 2**. Among 37 surgeries, only 1 perioperative complication occurred (2.7%, 95% CI 0.1%-14.2%), which was puncture site bleeding in a patient who used anticoagulants preoperatively due to pulmonary embolism. The patient had been bridged to low molecular weight heparin preoperatively, but hemostasis was not achieved after 5 minutes of compression, requiring suture hemostasis. The event was successfully handled intraoperatively without sequelae and was determined by the independent endpoint adjudication committee to be unrelated to the use of the 3D-printed measurement device.

**Table 2:** Perioperative and postoperative complications (n=37).

Complication type	Perioperative n (%)	Postoperative (30 days) n (%)	Total n (%)
Perioperative complications			
Puncture site bleeding	1 (2.7%)	—	1 (2.7%)
Pocket bleeding	0 (0%)	—	0 (0%)
Pneumothorax	0 (0%)	—	0 (0%)
Air embolism	0 (0%)	—	0 (0%)
Arrhythmia	0 (0%)	—	0 (0%)
Postoperative complications			
Infection	—	0 (0%)	0 (0%)
Catheter-related thrombosis	—	0 (0%)	0 (0%)
Subcutaneous hematoma	—	0 (0%)	0 (0%)
Poor wound healing	—	0 (0%)	0 (0%)
Catheter/Guidewire fracture	—	0 (0%)	0 (0%)
Port extrusion	—	0 (0%)	0 (0%)
Port rotation	—	0 (0%)	0 (0%)
Dysfunction	—	0 (0%)	0 (0%)
Total Complications	1 (2.7%)	0 (0%)	1 (2.7%)
Device-Related Adverse Events	0 (0%)	0 (0%)	0 (0%)

Perioperative and postoperative complications in 37 patients undergoing TIVAP placement with a 3D-printed depth measurement device. Perioperative complications were recorded intraoperatively by the surgeon. Postoperative complications were assessed at 30-day follow-up by independent blinded evaluators. The only perioperative complication (puncture site bleeding) occurred in a patient with preoperative anticoagulant use for pulmonary embolism and was successfully managed with sutures. No device-related adverse events were observed.

Successful first venous puncture was achieved in 28 patients, with a success rate of 75.7%. Nine patients (24.3%) required secondary puncture attempts or change of puncture site due to venous spasm or puncture failure. All adjustments were successfully completed without severe complications occurring. The novel 3D-printed measurement device was successfully applied in all 37 surgeries without any equipment failure, damage, or sterilization problems. The median measurement time of the device was 20 seconds (interquartile range 15–25 seconds), adding only negligible time to the overall surgical procedure. The device maintained structural integrity after experiencing multiple disinfection cycles during the study period, with no reports of probe deformation or wear.

No complications were observed during the 30-day postoperative follow-up period. Specifically, there were no cases of infection, catheter-related thrombosis, subcutaneous hematoma, poor wound healing, catheter/guidewire fracture, port extrusion, port rotation, or device dysfunction. Most importantly, no device-related adverse events occurred throughout the study period (0%, 95% CI 0%-8.1%), including no pocket laceration, vascular injury, measurement probe fracture, or infection introduced by the measurement device.

The total complication rate of 2.7% performed well compared with historical benchmarks. The latest systematic review by Huang et al. reported that the total complication rate range of traditional TIVAP implantation technology was 1.8%-14.4% (median 8.0%). The 2.7% observed in this study is comparable to the lower end of historical benchmarks. However, given the single-arm design of this early feasibility study, it is not possible to conclude that the 3D-printed device definitively reduces complications or is superior to conventional visual estimation methods. The zero device-related adverse event rate provides strong preliminary evidence for the safety of the 3D-printed depth measurement device used according to this standardized protocol.

### 3.3 Correlation between nutritional anthropometric indicators and pocket depth

The correlation analysis results between pocket depth and nutritional anthropometric indicators are shown in **Table 3**. Maximum abdominal fat thickness showed the strongest positive correlation with pocket depth (Pearson r = 0.803, 95% CI 0.64-0.89, *P* < 0.001), explaining 64.4% of the variance in pocket depth (R^2^ = 0.644). Waist circumference also showed strong positive correlation (*r* = 0.775, 95% CI 0.59-0.88, P <0.001), followed by BMI (*r* = 0.703, 95% CI 0.47-0.85, *P* < 0.001). Weight showed moderate positive correlation (*r* = 0.514, *P* = 0.001), while AST (*r* = -0.343, *P* = 0.038) and height (*r* = -0.334, *P* = 0.043) showed weak negative correlations. Pocket depth had no significant correlation with albumin, hemoglobin, or glucose levels (all *P >*0.05).

**Table 3:** Correlation analysis between pocket depth and clinical indicators (n=37).

Indicator	Correlation coefficient (r)	95% CI	P-value
Max abdominal fat thickness (mm)	0.803	0.64 to 0.89	<0.001
Waist circumference (cm)	0.775	0.60 to 0.88	<0.001
BMI (kg/m^2^)	0.703	0.49 to 0.84	<0.001
Weight (kg)	0.514	0.23 to 0.72	0.001
AST (U/L)	-0.343	-0.60 to -0.02	0.038
Height (cm)	-0.334	-0.59 to -0.01	0.043
Age (years)	-0.234	-0.52 to 0.10	0.163
Albumin (g/L)	0.047	-0.28 to 0.37	0.781
Hemoglobin (g/L)	-0.060	-0.38 to 0.27	0.722
Glucose (mmol/L)	-0.195	-0.49 to 0.14	0.248
Platelet count (×10^9^/L)	0.040	-0.29 to 0.36	0.813
INR	-0.093	-0.40 to 0.24	0.586

Pearson correlation coefficients were calculated to evaluate the relationship between pocket depth and various clinical indicators. Max abdominal fat thickness was measured using CT scanning at the thickest layer of abdominal wall fat. 95% CI, 95% confidence interval.

Simple linear regression analysis yielded the following prediction equation: Pocket Depth (mm) = 2.50 + 0.103 × Maximum Abdominal Fat Thickness (mm) (*β* = 0.103, SE = 0.013, *t* = 8.09, *P* < 0.001). It needs to be clarified that in clinical practice, operators routinely target a pocket depth of ≥5 mm to minimize port extrusion and infection risks. However, this equation reflects the anatomical limitations imposed by subcutaneous tissue availability rather than the surgical target. In patients with poor nutritional status and less subcutaneous fat, even with optimal surgical techniques, the achievable depth may still be limited to 2–3 mm. ROC curve analysis was used to determine the optimal cut-off value for predicting deep pocket (≥5 mm). Maximum abdominal fat thickness showed excellent predictive accuracy, with an optimism-corrected AUC of 0.942 (95% CI: 0.860-1.000) following bootstrap internal validation, and the optimal cut-off value was 14.5 mm (sensitivity 91.3%, specificity 85.7%) (**Figure 4**). Waist circumference and BMI also showed strong predictive value, with AUCs of 0.918 and 0.925, respectively.

**Figure 4:**
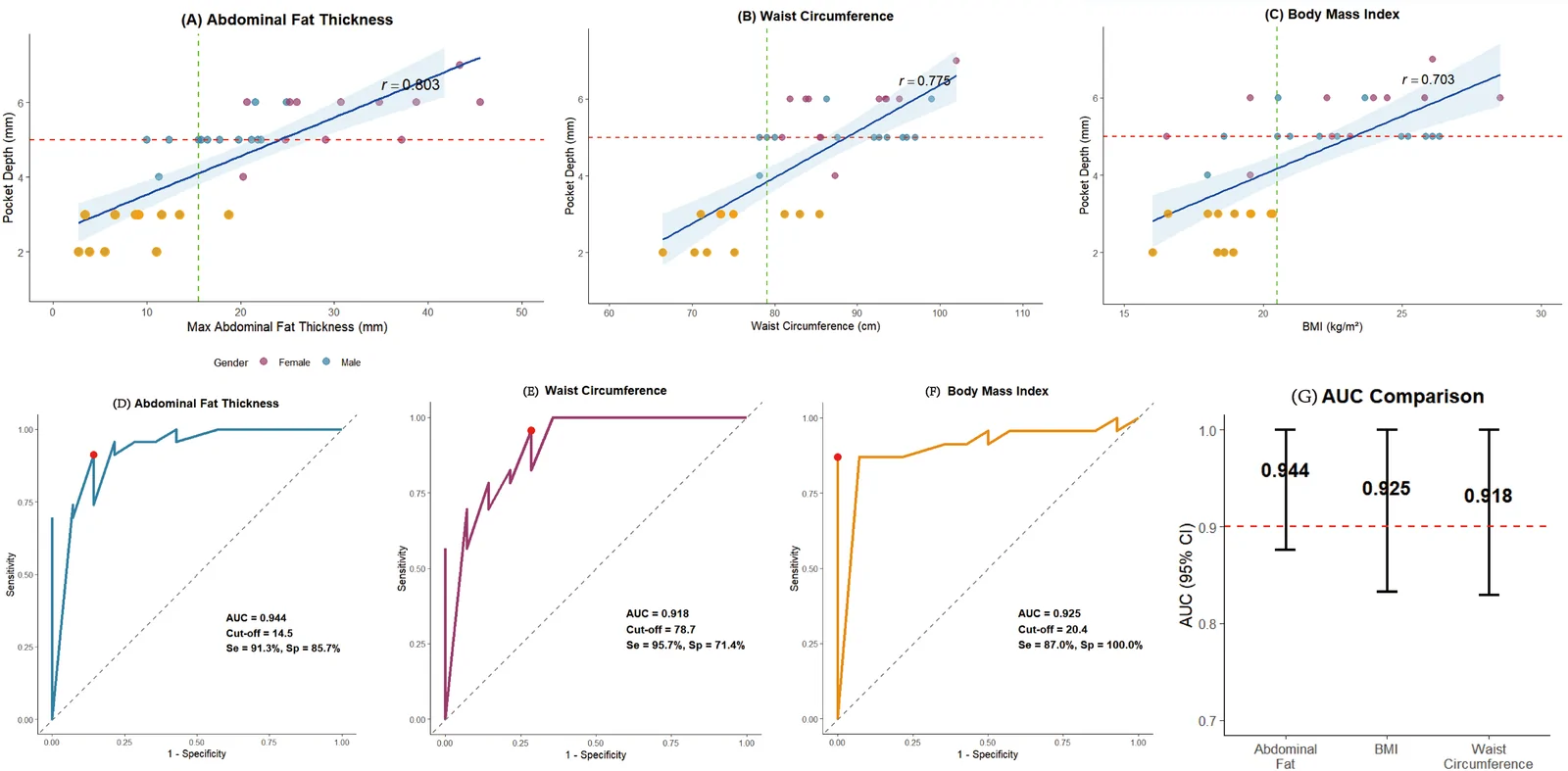
Correlation analysis between nutritional anthropometric indicators and pocket depth with prediction model for deep pocket placement. **(A–C)** Scatter plots showing significant positive correlations between max abdominal fat thickness (r = 0.803), waist circumference (r = 0.775), and BMI (r = 0.703) with pocket depth. Blue shaded areas represent 95% confidence intervals. Red dashed lines indicate the clinical target depth of 5mm. Green dashed lines represent optimal cut-off values derived from ROC analysis. **(D–F)** Receiver operating characteristic (ROC) curves demonstrating that max abdominal fat thickness has the highest predictive accuracy for deep pocket placement (≥5mm) with an apparent AUC of 0.944 and an optimism-corrected AUC of 0.942 (optimal cut-off 14.5mm), higher than that of waist circumference (AUC = 0.918) and BMI (AUC = 0.925). **(G)** Bar chart comparing AUC values further confirms the optimal predictive value of abdominal fat thickness. Stratified analysis revealed that 21.6% of patients (8/37) had depth limited to ≤3mm due to abdominal fat <10mm, indicating that subcutaneous tissue availability imposes anatomical constraints. The prediction model maintained consistency across different genders and implantation sites (arm port vs. chest wall port), supporting its potential as an exploratory indicator for identifying patients with anatomical constraints limiting pocket depth.

### 3.4 Stratified analysis by pocket depth

Patients were divided into five groups according to pocket depth for comparative analysis (**Table 4**). Maximum abdominal fat thickness (8.7 ± 4.7 vs 15.8 ± 6.4 vs 20.3 ± 7.2 vs 29.8 ± 8.4 vs 43.4 mm, *P* < 0.001), waist circumference (75.1 ± 5.5 vs 82.8 ± 6.4 vs 88.0 ± 6.9 vs 90.0 ± 6.0 vs 102.0 cm, *P* < 0.001), and BMI (18.6 ± 1.3 vs 18.8 ± 1.1 vs 22.7 ± 3.0 vs 23.8 ± 2.8 vs 26.1 kg/m^2^, *P* < 0.001) showed significant differences among depth strata. This dose-response relationship indicates that achievable pocket depth is subject to anatomical limitations of subcutaneous tissue availability. Among 8 patients (21.6%) with maximum abdominal fat thickness <10 mm, despite the surgical target of ≥5 mm, pocket depth was still limited to ≤3 mm. All 8 patients with abdominal fat <10 mm had pocket depth ≤3 mm. This finding highlights an important anatomical constraint: patients with poor nutritional status have limited subcutaneous tissue availability that restricts achievable pocket depth, regardless of surgical technique. We emphasize that no port extrusion events occurred in this 30-day cohort; therefore, the classification of these patients as “high-risk” is a theoretical extrapolation based on the established biomechanical principle that insufficient soft tissue coverage predisposes to device erosion, which requires confirmation through long-term follow-up.

**Table 4:** Stratified analysis by pocket depth (n=37).

Pocket depth	n	Waist circumference (cm)	Max abdominal fat thickness (mm)	BMI (kg/m^2^)
≤3mm	12	75.1 ± 5.5	8.7 ± 4.7	18.6 ± 1.3
4mm	2	82.8 ± 6.4	15.8 ± 6.4	18.8 ± 1.1
5mm	13	88.0 ± 6.9	20.3 ± 7.2	22.7 ± 3.0
6mm	9	90.0 ± 6.0	29.8 ± 8.4	23.8 ± 2.8
≥7mm	1	102.0	43.4	26.1
P-value	-	<0.001	<0.001	<0.001

Patients were stratified into five groups based on pocket depth measurements. Continuous variables are presented as mean ± standard deviation. P-values were calculated using one-way ANOVA for trend across depth strata.

Based on these findings, we propose an exploratory, hypothesis-generating risk stratification framework for patient counseling and planning: maximum abdominal fat thickness <10 mm → shallow pocket high risk (achievable ≤3 mm); 10–25 mm → moderate risk (achievable 4–5 mm); >25 mm → low risk (achievable ≥6 mm). This stratification does not recommend malnourished patients to accept shallower pockets; rather, it identifies patients who may benefit from enhanced postoperative monitoring or require nutritional optimization. Gender subgroup analysis showed consistent correlation patterns; male patients (*n* = 25) maintained strong correlation between maximum abdominal fat thickness and pocket depth (r = 0.805, *P* < 0.001). TIVAP type (arm port *n* = 30 vs chest wall port *n* = 7) had no significant effect on the correlation between nutritional indicators and pocket depth (interaction *P* = 0.685), suggesting that the prediction model is applicable to different implantation sites.

## 4 Discussion

This study is a prospective single-arm clinical trial that for the first time evaluated the safety and feasibility of a 3D-printed pocket depth measurement device in TIVAP implantation and explored the quantitative association between preoperative nutritional anthropometric indicators and achievable pocket depth. Main findings include: (1) the measurement device demonstrated an acceptable preliminary safety profile in this early feasibility setting, with a device-related adverse event incidence rate of 0%; (2) maximum abdominal fat thickness showed an extremely strong positive correlation with pocket depth (*r* = 0.803), explaining 64.4% of depth variance; (3) the nutrition indicator-based prediction model could identify 21.6% of patients with abdominal fat <10 mm, all of whom had achievable depth limited to ≤3 mm due to anatomical limitations. These findings provide new evidence-based basis for individualized planning of TIVAP implantation and reveal the decisive influence of subcutaneous tissue reserves on surgical anatomical conditions from the perspective of nutritional anthropometry.

The total complication rate observed in this study was 2.7%, comparable to the lower end of the range (1.8%-14.4%, median 8.0%) reported in previous systematic reviews (4). This may be attributed to multi-factor synergistic effects: first, standardized pocket depth measurement aimed to minimize variability in depth assessment; secondly, the modified zero-tunnel oblique incision technique avoided subcutaneous tunnel injury of the traditional double-incision technique, reducing overall surgical time (2); thirdly, layered cosmetic suturing technology optimized skin edge alignment, reducing incision tension-related poor healing (9–11). Notably, the only perioperative complication that occurred in this study (puncture site bleeding) was related to the patient’s preoperative anticoagulant status and was determined by the independent committee to be unrelated to the measurement device, further supporting the safety of the device. However, given the single-arm design of this early feasibility study, it is not possible to conclude that the 3D-printed device actually reduces complication rates or is superior to conventional visual estimation methods.

Regarding the correlation between nutritional indicators and pocket depth, this study for the first time quantitatively reported the strong association between maximum abdominal fat thickness and achievable depth (*r* = 0.803, R^2^ = 0.644). This finding is consistent with the value of CT body composition analysis in nutritional assessment of cancer patients emphasized by ESPEN guidelines (5). Traditional nutritional indicators such as BMI, although correlated with depth (*r* = 0.703), cannot distinguish between muscle and fat distribution, which may lead to assessment bias in cachectic or sarcopenic patients. The CT-measured maximum abdominal fat thickness method adopted in this study can more accurately reflect subcutaneous fat reserves, providing an objective basis for preoperative anatomical condition assessment. Different from previous studies that only focused on postoperative complications, this study established a direct association between nutritional status and intraoperative anatomical limitations, filling the evidence gap in this field.

The clinical significance of this study is mainly reflected in three levels. First, the 3D-printed measurement device achieved objective quantification of intraoperative depth, solving the clinical pain point of long-term reliance on operator experience estimation. The measurement process took an average of only 20–30 seconds and did not significantly increase the total surgical time, having good clinical operability. Secondly, the exploratory thresholds based on abdominal fat thickness (optimism-corrected AUC = 0.942) may help identify patients with anatomical constraints. In this cohort, all 8 patients (21.6%) with subcutaneous fat <10 mm had depth limited to ≤3 mm; such patients have anatomical limitations that are difficult to overcome even with optimal surgical techniques. Preoperative identification of such patients helps to: (1) fully inform patients and family members of complication risks; (2) guide patients in preoperative and postoperative nutritional optimization interventions; (3) enhance postoperative follow-up monitoring. Thirdly, the exploratory risk stratification framework proposed in this study (<10 mm, 10–25 mm, >25 mm) provides a concise hypothesis-generating framework. It should be noted that while 14.5 mm is the statistically optimal cut-off derived from the Youden index, the three-tier stratification was pragmatically defined based on the observed distribution of achievable depths (≤3 mm, 4–5 mm, ≥6 mm) in our cohort to offer a clinically actionable framework, rather than serving as an absolute diagnostic gold standard.

Thinking deeply from a nutritional and technological perspective, this study reveals the direct impact of nutritional heterogeneity on surgical anatomical conditions. The integration of 3D-printed measurement tools and body composition analysis extends beyond oncology; it provides a methodological reference for other specialties dealing with subcutaneously implanted devices (such as cardiology for pacemakers or endocrinology for insulin pumps). This broader applicability enhances the relevance of our findings beyond specialized surgical fields, calling for clinicians across various disciplines to pay attention to body composition analysis, especially subcutaneous fat reserve assessment, in addition to focusing on traditional indicators such as weight and BMI, to achieve more precise treatment planning.

This study has several limitations that need to be objectively explained. First, the sample size was relatively small (n = 37). Although this meets FDA recommendations for early feasibility medical device studies to identify primary safety signals, the findings remain preliminary. While 1,000-iteration bootstrap internal validation demonstrated robust predictive performance (optimism-corrected AUC = 0.942, with a minimal optimism bias of 0.003), the reported cut-off values and risk stratification thresholds are still exploratory and hypothesis-generating, requiring rigorous external validation in larger, multicenter cohorts before clinical implementation. Second, all pocket depth measurements were performed by a single surgeon. While this approach minimized intraoperative variability, it precludes formal assessment of interobserver reliability, representing a methodological limitation. Third, the primary safety endpoint was 30 days postoperatively; 3-month and long-term follow-up data are still being collected, and the incidence rates of late complications (such as port extrusion, catheter fracture) need to be reported subsequently. Fourth, although we validated the povidone-iodine disinfection protocol through bacterial culture and precision testing, we acknowledge its limitations (1): it relies on strict adherence to the 30-minute immersion time (2); long-term material degradation beyond 20 cycles was not evaluated (3); the protocol has not been validated against microbiological endpoints in actual clinical reuse; and (4) chemical disinfection cannot achieve absolute sterility. Future studies should monitor infection rates associated with device reuse. Fifth, the chest wall port sample size was small (n = 7), with limited subgroup analysis statistical power; differences in depth prediction models among different implantation sites need further verification. Sixth, this study was not registered in a public clinical trial registry prior to enrollment; while appropriate for an early single-center feasibility assessment of a measurement device, future interventional trials should ensure prospective registration. Finally, the prediction model relied on abdominal fat thickness as a surrogate for systemic nutritional status rather than direct local subcutaneous thickness at the implantation site. Future studies should incorporate high-resolution ultrasound to directly measure local subcutaneous tissue thickness at the arm or chest wall to further refine the predictive accuracy.

Based on the results of this study, future research could focus on the following directions (1): multicenter external validation of the prediction model established in this study to determine the universality of optimal cut-off values (2); conduct randomized controlled trials to compare complication differences between 3D-printed measurement device-assisted surgery and traditional visual estimation surgery (3); explore the impact of preoperative nutritional intervention (such as oral nutritional supplementation) on improving subcutaneous fat reserves and achievable depth (4); develop ultrasound measurement alternative solutions for abdominal fat thickness to reduce CT radiation exposure (5); long-term follow-up (six months and above) to evaluate the dose-response relationship between pocket depth and late complications (port extrusion, infection).

## 5 Conclusion

In the context of an early feasibility study, this research demonstrated the preliminary safety and feasibility of the 3D-printed pocket depth measurement device in TIVAP implantation, with zero device-related adverse events observed as primary safety signals. For the first time, it quantified the strong correlation between nutritional indicators (particularly maximum abdominal fat thickness) and pocket depth, revealing the impact of subcutaneous tissue reserves on surgical anatomical limitations. The exploratory thresholds generated accordingly may help identify patients with anatomical limitations associated with shallow pocket depth, guide individualized planning, and provide a new nutritional perspective for subcutaneously implanted devices.

## Data Availability

The original contributions presented in the study are included in the article/supplementary material. Further inquiries can be directed to the corresponding authors.
